# Supplementary material for: Analysis of the progression of cervical cancer in a low-and-middle-income country: From pre-malignancy to invasive disease
Source: Tumour Virus Res. 2024 Dec 12;19:200299. doi: 10.1016/j.tvr.2024.200299 (PMC11729683; doi:10.1016/j.tvr.2024.200299)
Supplement: Multimedia component 2 [file mmc2.docx]

**Supplemental figures**

1. **S1. Diagram/flow chart of what happened to all the samples**
2. **S2 –insert S3 HPV31 sublineage figures here probably–**
3. **S3 DNA repair genes**


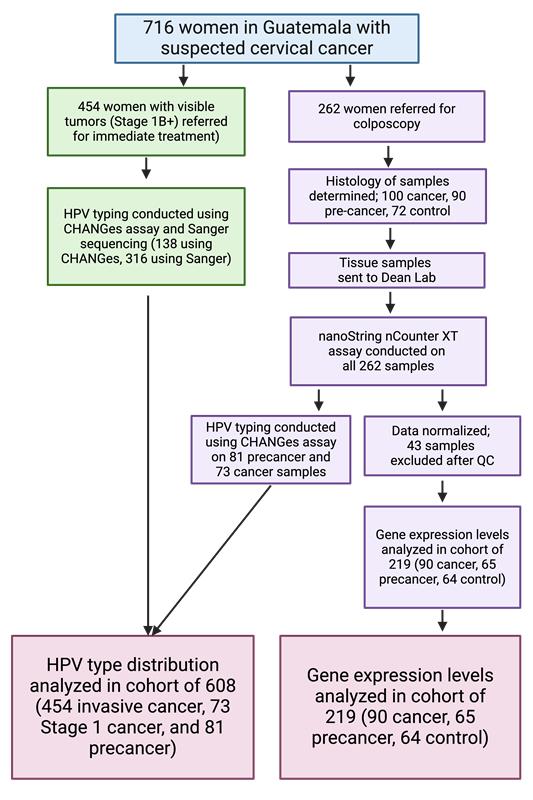


**Figure S1. Sample flowthrough and exclusions.**

**
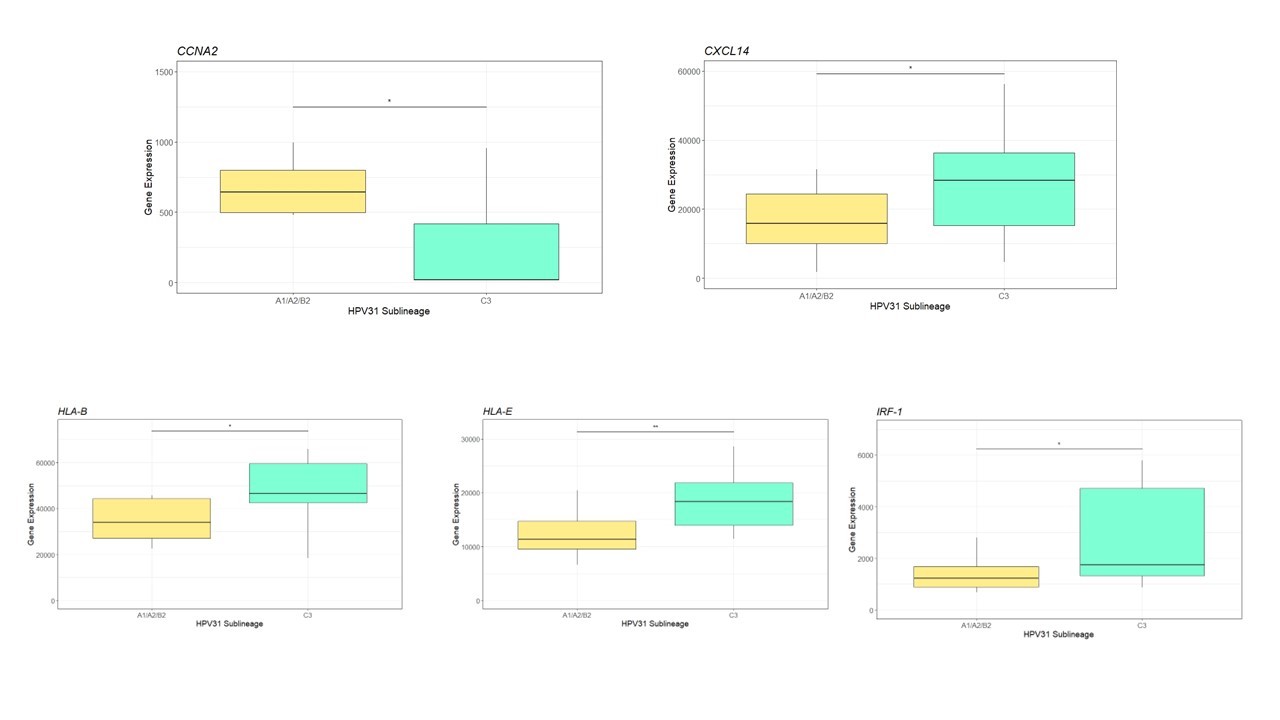
**

**Supplementary Fig. S2. Differences in gene expression between HPV31-C3-positive precancers and HPV31-positive precancers of other sublineages.** Box and whisker plots representing the expression level of genes differentially expressed in HPV31-C3-positive precancers and HPV31-positive precancers of other sublineages. Gene expression is shown on the y-axis, while HPV type is on the x-axis. The expression of *CCNA2* is lower in HPV31-C3-positive precancers, while the expression of *CXCL14*, *IRF-1*, *HLA-B*, and *HLA-E* is higher in HPV31-C3-positive precancers.


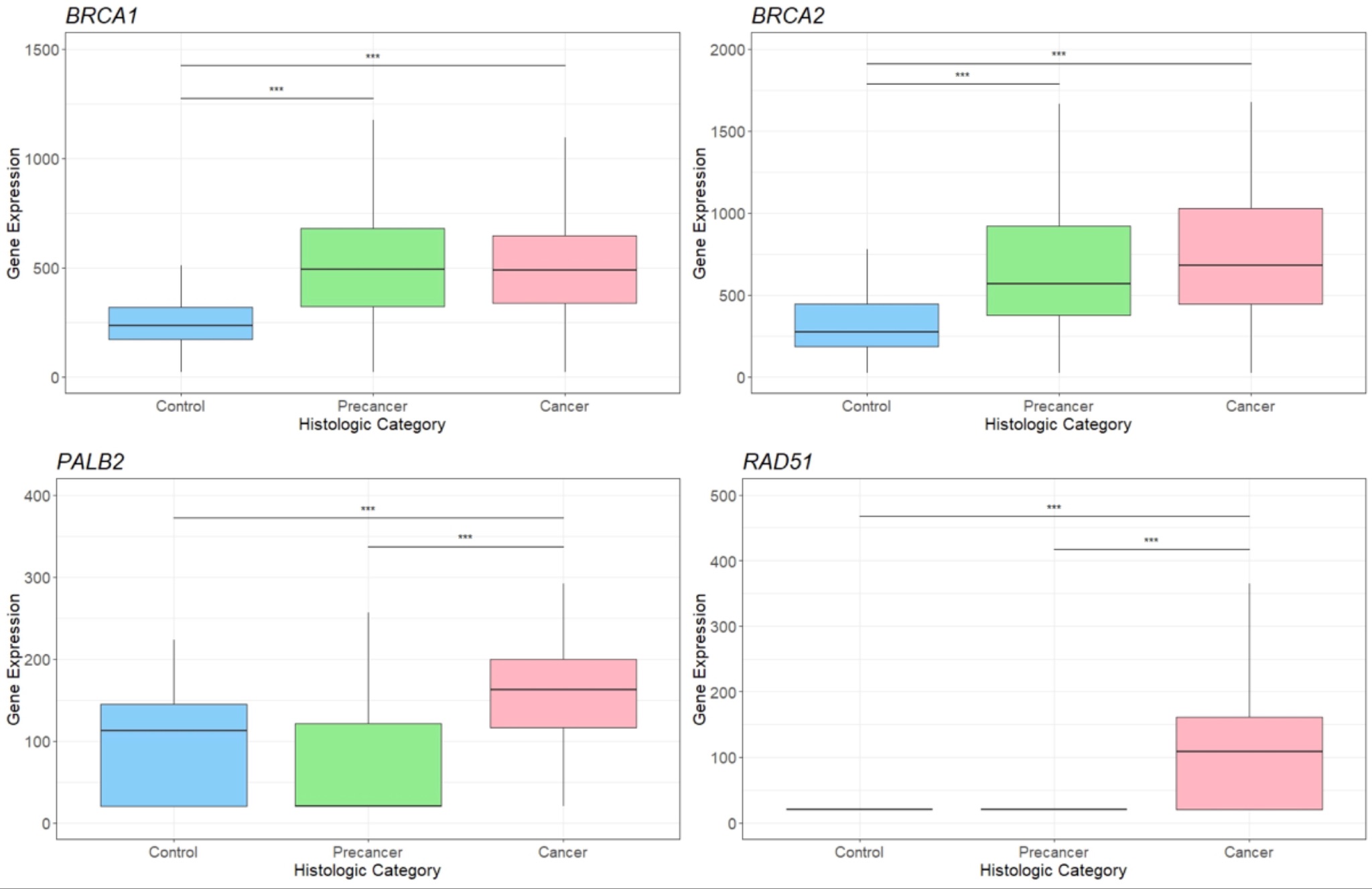


**Supplementary Fig. S3. Analysis of DNA repair gene expression in controls, precancer, and cancer.** These box and whisker plots represent the expression level of DNA repair genes corresponding to their histological category. Gene expression is shown on the y-axis, while the histological type is shown on the x-axis. The expression of *BRCA1* and *BRCA2* increases in cancer and precancers compared to the control group. The expression of *PALB2* and *RAD51* increases in cancer compared to precancer and controls. *** indicates significance of p < 0.001, ** indicates significance of p < 0.01, and * indicates significance of p < 0.05.
